# Progress in Cardiac Magnetic Resonance Feature Tracking for Evaluating Myocardial Strain in Type-2 Diabetes Mellitus

**DOI:** 10.2174/0115733998277127231211063107

**Published:** 2024-01-16

**Authors:** Li-Ting Shen, Rui Shi, Zhi-Gang Yang, Yue Gao, Yi-Ning Jiang, Han Fang, Chen-Yan Min, Yuan Li

**Affiliations:** 1 Department of Radiology, West China Hospital, Sichuan University, Chengdu, Sichuan, China

**Keywords:** Cardiac magnetic resonance feature tracking, myocardial strain, type-2 diabetes mellitus, cardiovascular disease, quantitative cardiovascular system, heart failure

## Abstract

The global prevalence of type-2 diabetes mellitus (T2DM) has caused harm to human health and economies. Cardiovascular disease is one main cause of T2DM mortality. Increased prevalence of diabetes and associated heart failure (HF) is common in older populations, so accurately evaluating heart-related injury and T2DM risk factors and conducting early intervention are important. Quantitative cardiovascular system imaging assessments, including functional imaging during cardiovascular disease treatment, are also important. The left-ventricular ejection fraction (LVEF) has been traditionally used to monitor cardiac function; it is often preserved or increased in early T2DM, but subclinical heart deformation and dysfunction can occur. Myocardial strains are sensitive to global and regional heart dysfunction in subclinical T2DM. Cardiac magnetic resonance feature-tracking technology (CMR-FT) can visualize and quantify strain and identify subclinical myocardial injury for early management, especially with preserved LVEF. Meanwhile, CMR-FT can be used to evaluate the multiple cardiac chambers involvement mediated by T2DM and the coexistence of complications. This review discusses CMR-FT principles, clinical applications, and research progress in the evaluation of myocardial strain in T2DM.

## 1 INTRODUCTION

**Fig. (1):**
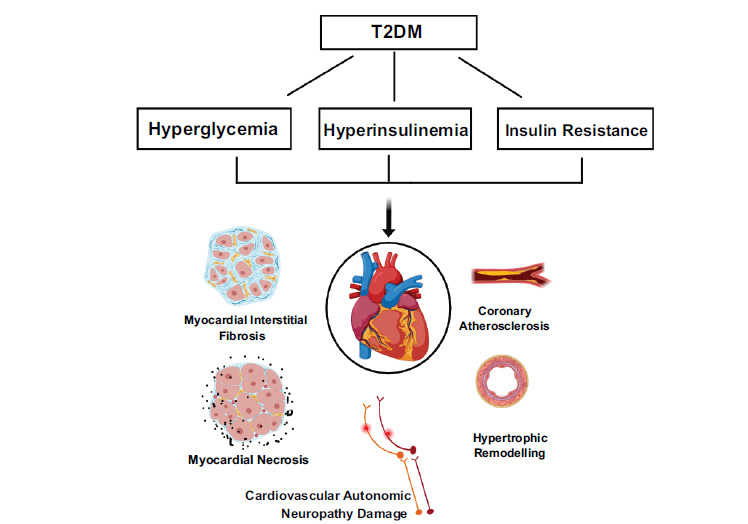
Pathophysiology of diabetes-related cardiac injury (**Source:** Figure prepared by the author using MedPeer software).

The International Diabetes Federation Diabetes Atlas 10^th^ edition confirms that diabetes mellitus (DM) is one of the fastest-growing global health emergencies of the 21^st^ century [1]. Cardiovascular-related complications account for 52% of mortalities in type-2 diabetes mellitus (T2DM) and 44% in type-1 DM [2, 3]. The pathophysiology of DM-related cardiac injury is multifactorial and complex, ultimately leading to cardiac remodeling and heart failure (HF) through several metabolic pathways [4-7]. One pathway is direct damage to myocardial cells and interstitium caused by persistent hyperglycemia, hyperinsulinemia or hypoinsulinemia, alteration in fatty-acid metabolism, impaired calcium homeostasis, and inflammation. Eventually, myocardial interstitial fibrosis, mitochondrial lysis, cardiomyocyte apoptosis, and necrosis occur [8-11]. Another pathway is damage to heart-related blood vessels and nerves. Coronary microvascular hyperplasia and fibrosis are the most common of these findings in DM. Hyperglycemia induces endothelial dysfunction, oxidative stress, and reduced nitric oxide production, which lead to myocardial ischemia and hypoxia, induce myocardial cell apoptosis, form tiny necrotic lesions and scar tissue, and eventually lead to cardiac remodeling [12]. In addition, DM-associated cardiovascular autonomic neuropathy dysfunction is related to abnormal heart rate and vascular dynamics (Fig. **1**) [13, 14].

Most patients with T2DM have only asymptomatic left ventricle (LV) diastolic dysfunction without obvious structural changes initially. With progression, LV hypertrophy results in diastolic filling impairment, prolonged isovolumic relaxation, and increased LV end-diastolic pressure that precedes the development of systolic dysfunction [15]. Traditional heart-function parameters, such as left-ventricular ejection fraction (LVEF), have limitations in the diagnosis of early cardiomyopathy in patients with T2DM, especially for those in the preserved LVEF stage [16-19]. Myocardial strain parameters, considered to be sensitive indicators of cardiac insufficiency, can be used to evaluate the global and regional deformation and mechanical characteristics of the myocardium [20, 21]. CMR-FT can provide abundant myocardial strain parameters with good repeatability, such as peak strain (PS), strain rate, and LV torsion [22]. They are all sensitive indicators of LV function, which have been proven to be abnormal in patients with asymptomatic T2DM [22, 23]. CMR-FT has also made progress in evaluating left and right ventricular strain, as well as the atrioventricular and biventricular interaction effects. Therefore, myocardial strain analysis by CMR-FT can detect subclinical cardiac disorders and monitor therapeutic efficacy in T2DM patients thoroughly and accurately.

## 2 PRINCIPLE OF CMR-FT

**Fig. (2):**
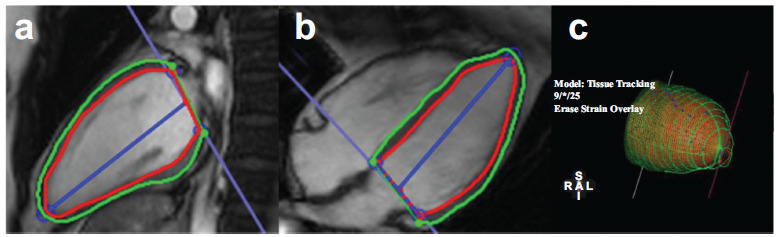
Analysis of LV strain by cardiac magnetic resonance cine images. The LV endocardium (red) and epicardium (green) were outlined on the LV two-chamber long axis (**a**) and four-chamber long axis (**b**) images of end-diastolic. (**c**) The 3D volume tissue tracking model of LV automatically established (**Source:** Figure prepared by the author using the CVI42 post-processing software).

CMR is the gold standard for evaluating cardiac structure and function and has the advantages of one-stop, non-radiation, non-invasive, accurate quantitative assessment of cardiac structure, function, and perfusion [24]. The feature-tracking technology used in CMR is based on a standard steady-state, free precession, and cine imaging sequence, enabling rapid and accurate assessment of myocardial strain. CMR-FT recognizes anatomical voxels along the cavity–myocardial boundary by applying methods of maximum likelihood of interest between two frames and then identifies the image features through the optical flow method and tracks these features on the continuous image sequence of the entire cardiac cycle [25, 26]. With this, the target myocardium displacement can be accurately measured, and the strain and strain rate can be calculated to reflect the systolic and diastolic functions [27]. Post-processing software is used to manually trace the endocardial and epicardial borders at the end diastole, which then automatically calculates the strain values (Fig. **2**).

**Fig. (3):**
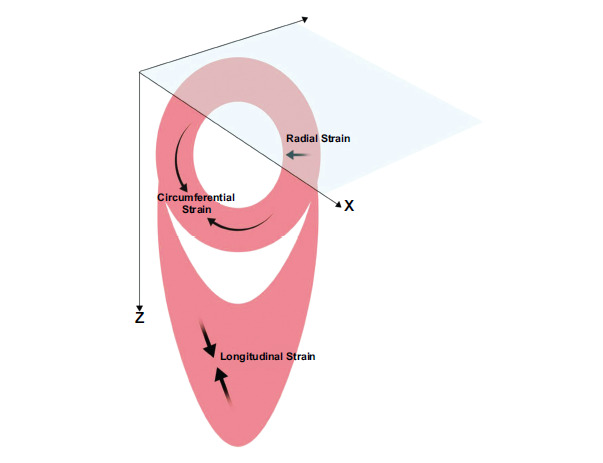
The diagram of strain in three directions of the left ventricle (**Source:** Figure prepared by the author using MedPeer software).

The myocardial strain deformation index is defined as the ratio of dimensional changes from resting state (end-diastolic) to systolic state (end-systolic) [28]. Considering that in a myocardial fiber, L0 is its initial length in end diastole and L1 is its final length in end systole, myocardial strain (S) can be defined as follows: S = (L1−L0)/L0 [20]. Due to the three layers of cardiac muscle, there are three strain directions: Longitudinal, radial, and circumferential (Fig. **3**). Strain rate (SR) represents the time derivative of strain values and can reflect regional diastolic dysfunction efficiently [22]. Torsion is a wringing motion that assists in ejecting blood from the LV during the systolic phase of each cardiac cycle. Torsion is directly related to the myofiber orientation and function, and can also be used to discriminate subtle changes in systolic and diastolic dysfunction in the setting of a variety of cardiomyopathic states [28].

## 3 THE COMPARISON OF CMR-FT WITH CMR-TAGGING AND SPECKLE-TRACKING ECHOCARDIOGRAPHY (STE)

Measurement of myocardial strain can be performed by using a variety of imaging methods, including speckle-tracking echocardiography (STE), cardiac magnetic resonance (CMR)-tagging (CMR-TAG), and cardiac magnetic resonance feature-tracking technology (CMR-FT) [29, 30]. They have their own strengths and limitations. STE has a relatively high temporal resolution, good spatial resolution, and low signal-to-noise ratio. However, STE can lead to a decrease in accuracy and repeatability due to its great dependence on image quality [31, 32]. CMR tagging allows quantitative measurement of regional intramyocardial motion measures, but its clinical utility is hampered by the need to acquire additional sequences, time-consuming post-processing, and diastolic tag fading [33, 34]. CMR-FT provides the most accurate and reproducible assessments of global atrial and ventricular volumes and function available. It does not require specific encoding pulses and greatly reduces post-processing time, so the clinical use of CMR-FT has good prospects [34].

Several studies have evaluated the reproducibility of CMR-FT (using CMR-tagging as a reference standard) and found a clear correlation between CMR-FT and CMR-tagging for basic strain parameters (peak strain of the septum, ICC: 0.58, R: 0.55; lateral wall peak strain, ICC:0.54, R:0.59) [34]. This means the agreement between CMR-tagging and CMR-FT is high. Meanwhile, the uniformity has been found to be higher for CMR-tagging and CMR-FT than for CMR-TAG and STE. This applies to ICC values, Bland–Altman characteristics, and the correlation coefficient (R) [33, 34]. In addition, a large number of studies involving children and adults have compared CMR-FT with CMR-TAG and STE in congenital and acquired heart disease, with all showing great correlation [35]. The CMR-FT technique makes multi-dimensional and quantitative evaluation possible and provides accurate information for early functional diagnosis of heart injury in T2DM patients.

## 4 EVALUATION BY CMR-FT OF MYOCARDIAL STRUCTURE AND FUNCTION IN PATIENTS WITH T2DM

A large number of patients with T2DM do not have overt clinical signs, such as myocardial ischemia and HF, but the hearts of diabetics already have potential dysfunction. Pathological manifestations are reduced shortening of longitudinal and circumferential muscle fibers and/or problems of peripheral myocardial fiber systolic dysfunction. Studies have shown that one-sixth of patients with T2DM do not have myocardial ischemia, but they show a combined decrease in global longitudinal strain (GLS) and global circumferential strain (GCS) of the LV, which may be a sign of HF [36, 37]. In the population without cardiovascular disease, elevated HbA1c levels were independently associated with impaired LV diastolic function (GCPS: *β* = −0.203, *p* = 0.023; GLPS: *β* = −0.207, *p* = 0.040) [38]. It is suggested that CMR-FT has great potential to monitor early myocardial systolic and diastolic dysfunction in T2DM patients.

### 4.1 Evaluation of LV Structure and Function

Several studies have confirmed that early detection of LV strain is of great significance for intervention and delay of myocardial deterioration in patients with T2DM. Conventional cardiac indicators, such as LV end-diastolic volume (LVEDV) and LV end-systolic volume (LVESV), have not been found to be different between the diabetic group with preserved LVEF and control group, indicating that traditional CMR functional parameters cannot be reliably used to monitor subclinical changes in diabetic hearts [39, 40]. Strain parameters determined by CMR-FT can provide functional information for the early identification of cardiac damage in T2DM patients. Previous studies have suggested GLS to be lower in patients with DM than in the control group [41]. The reason may be that DM-related myocardial diseases predominantly cause subendocardial dysfunction in the early stage of the disease, leading to changes in the longitudinal LV mechanics. Advanced damage to the circumferential and radial muscle fibers occurs, indicating complete involvement of the myocardium, leading to combined systolic and diastolic dysfunction [42, 43].

One study indicated that asymptomatic patients with T2DM had early-stage LV systolic and diastolic subclinical dysfunction with global longitudinal and circumferential strain impairments, and after 2 years of follow-up, these patients experienced further decline in LV global longitudinal and circumferential strain and increase in LV mass despite no clinical symptoms [44]. Xie *et al*. also explored the relationship between LV strain parameters and LV remodeling index (LVMVR), and found LV GLS to be independently associated with LVMVR (*β* = 0.297; *P* = 0.014). LVEF was positively correlated with the global radial strain (GRS) (*r* = 0.688, *P* < 0.001), GCS (*r* = 0.725, *P* < 0.001), and GLS (*r* = 0.415, *P* < 0.001) [45]. In a meta-analysis, LV GLS was found to predict cardiovascular events more reliably than LVEF, and the decrease in LV GLS occurred earlier and more significantly than shown by LVEF [46]. In addition, studies have found that LV PS is independently associated with all-cause mortality, and the assessment of LV GLS is beneficial for reducing the risk of 5-years mortality in patients with diabetes [23]. Reduced GLS of the LV is an important predictor of adverse cardiac events (myocardial infarction, new-onset angina, and heart failure) in patients with DM [46].

### 4.2 Evaluation of Left-atrial Structure and Function

In recent years, left atrial (LA) remodeling has gained much attention because a large amount of evidence has shown the predictive value of LA enlargement (LAE), function, and mechanics estimated by CMR-FT in patients with T2DM. Studies have shown that many patients with T2DM had impaired LA strain and LAE, but no LV hypertrophy. Cameli *et al*. showed that LA strain injury appeared even earlier than LV strain in the early stages of diabetic cardiomyopathy. As an early diagnostic parameter, LA strain may be more sensitive than the LV strain for early detection of myocardial abnormalities in patients with DM [47]. One possible explanation has to do with anatomy; the LA has a very thin, single-layered wall that is very sensitive to subtle stimuli and injuries. Furthermore, LA function is independently associated with HF-related hospitalization and mortality, and LA strain is a sensitive marker of LV diastolic dysfunction [48-50].

LA fibrosis in patients with DM may be the cause of LA remodeling and atrial fibrillation (AF) because increased collagen deposition can slow atrial conduction velocity and generate splitting of the propagating wavefront, leading to reentry [50]. Patients with DM can also develop hypertension through insulin resistance, resulting in impaired LA function. DM can also induce conduction and refractory disorders that promote LA remodeling and dysfunction. These changes may induce paroxysmal AF, further induce LAE and systolic dysfunction, and further deterioration may lead to persistent AF, forming a vicious cycle of LA function deterioration [51].

**Fig. (4):**
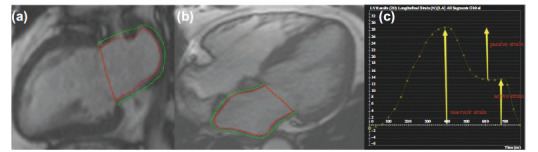
Analysis of LA strain by cardiac magnetic resonance cine images. The LA endocardium (red) and epicardium (green) have been outlined on the LV two-chamber long axis (**a**) and four-chamber long axis (**b**) images of end-diastolic. (**c**) Left atrial myocardial longitudinal strain curve (**Source:** Figure prepared by the author using the CVI42 post-processing software).

The phasic LA function has three phases: Reservoir during systole, conduit during early diastole, and booster pump during late diastole. The reservoir function depends on LA relaxation and compliance regulated by LV systolic function; the conduit function is related to LV diastolic function, which depends on LV relaxation and chamber stiffness, while the contractile function represents intrinsic LA contractility and LV end-diastolic compliance and pressure (Fig. **4**) [52]. Studies have shown that the LA reservoir and conduit function assessed by CMR-FT are associated with hospitalization or death due to HF. The investigators of the Multi-Ethnic Study of Atherosclerosis showed that the LA total and passive- and active-emptying fractions were independently associated with incident cardiovascular disease in a diabetic multi-ethnic population free of any clinically recognized cardiovascular disease at baseline [53-55].

### 4.3 Evaluation of Right-ventricular Structure and Function

Due to the increasing recognition of the prognostic role of right-ventricular (RV) function in various cardiovascular and pulmonary diseases, RV dysfunction is an important indicator of cardiac morbidity and mortality in various cardiovascular diseases [56]. Therefore, research on RV strain examined by echocardiography or CMR has been greatly improved, especially for assessing RV longitudinal strain. However, the quantitative assessment of RV function by echocardiography is limited due to the RV’s complex geometry, dense trabeculae, special retrosternal position, and complex motion. CMR-FT has the advantages of high image resolution and short processing time as well as gradually being more widely used. This technology can provide RV longitudinal and circumferential motion information of high repeatability [57].

The pathological mechanism of LV in DM is also applicable to RV because of the systematic nature of the disease. The RV myocardium wall mainly comprises longitudinal myofiber; thus, RV-impaired longitudinal strain and strain rate can indicate RV longitudinal systolic and diastolic dysfunction [58]. Numerous studies have confirmed that patients with DM have lower RV GLS than non-patients with DM. In a prospective trial, RV GLS and GCS were significantly lower in patients with DM with and without reduced RVEF than in healthy controls, and RV GLS was an independent factor of RV dysfunction [59]. Linssen *et al*. found no correlation between RV systolic and diastolic function and LV function in patients with DM, suggesting that DM may directly impair RV function [60]. Therefore, assessment of RV function and structure based on CMR-FT technology is valuable for a comprehensive understanding of heart injury in patients with T2DM.

### 4.4 Evaluation of Left-atrioventricular Coupling Effect

Anatomically, the LV and LA are connected by the left-atrioventricular orifice, and the LV produces a direct mechanical stretch on the LA during systole; functionally, the LA afterload depends on the LV end-diastolic pressure and LV wall stiffness; the function of LA storage period ensures that LV can be filled quickly under short-term and low-load conditions, which is very important for situations with rapid heart rate, such as exercise. Mechanically, LA systolic strain increases with LV end-diastole; LA storage strain is closely related to longitudinal shortening of LV myocardium, and a decrease in LA reservoir strain often indicates LV systolic dysfunction [61]. Clinically, some diseases that cause LV hypertrophy, such as hypertension and aortic stenosis, can accelerate LA remodeling by affecting LV diastolic function [62]. Those findings suggest that the LV function has a great influence on the LA, and LA remodeling and dysfunction are the reflection of LV diseases, such as hypertension, HF, cardiomyopathy, and others.

Given that T2DM is an important cause of atrioventricular dysfunction, it is essential to evaluate the atrioventricular effect on myocardial injury in patients with T2DM. Previous studies on diabetic populations focused on LV structure and function, but atrioventricular coupling has been shown to be an important predictor of cardiovascular morbidity and mortality in those patients because each LA function phase has a decisive role in the atrioventricular coupling mechanism [63]. Shao *et al*. concluded LA strain to be significantly higher in patients with DM and hypertension than in patients with hypertension but without DM [47]. It is speculated that hypertension hinders blood flow from LA to LV by increasing LV myocardial stiffness. The increased LA preload stimulates the Frank–Starling mechanism of LA to complete the compensation within a certain range. Many studies have shown that LV GLS (*r* values: 0.64 and 0.51, respectively) and LV filling pressure (*r* values: −0.52 and −0.57, respectively) are the main determinants of LA reservoir and contractile strain. LVEDV is an independent factor affecting LA reservoir strain, and LVESV is an independent factor affecting LA contractile strain [64, 65]. Therefore, assessment of the left atrium–ventricular interaction effect in patients with T2DM more accurately characterizes cardiac injury.

### 4.5 Evaluation of Interventricular Interaction Effect

**Fig. (5):**
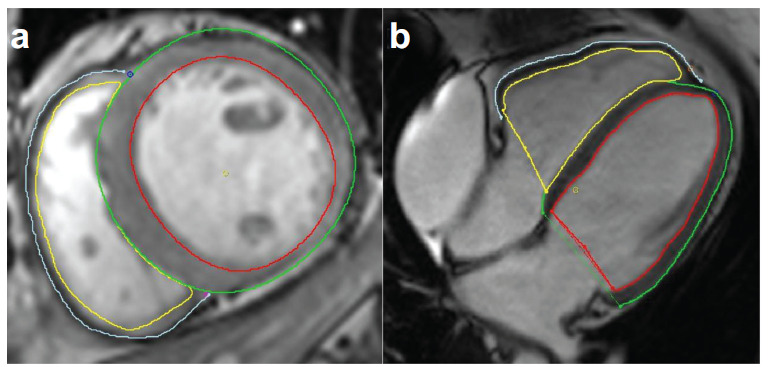
Analysis of LV and RV strain by cardiac magnetic resonance cine images. The biventricular endocardium and epicardium are outlined on the short-axis images (**a**) and four-chamber long-axis images (**b**) (**Source:** Figure prepared by the author using the CVI42 post-processing software).

Interventricular interaction is defined as the transmission of force from one ventricle to the other through the myocardium and pericardium, independent of neural, humoral, and circulatory effects. The interaction between ventricles is due to their close anatomical relationship. They are surrounded by common myocardial fibers and the same pericardial cavity, and they have a common interventricular septum (IVS) (Fig. **5**) [66]. Many studies have shown that in isolated hearts with the absent pericardium, the independent load of one ventricle shifts the diastolic pressure–volume curve of the contralateral ventricle upward and to the left, indicating that when the interventricular circulatory connection is disrupted, there is still mechanical action [67].

An animal experiment showed that about 20%–40% of the RV output is related to the LV systolic effect. We speculate that T2DM not only directly impairs the RV function, but also leads to RV dysfunction by impairing the function of LV and IVS. In one study, the PS of RV in hypertensive subjects was closely related to that of LV and interventricular-septum segmental strain. In a diabetic group with hypertension, the reduction of RV GLS was related to LV GLS damage [68]. Todo S *et al*. used RV free-wall tension to study RV systolic dysfunction and its relationship with LV longitudinal myocardial dysfunction in patients with T2DM with preserved LVEF and found that RV subclinical systolic dysfunction was associated with LV longitudinal PS [45]. In another study, higher levels of epicardial adipose tissue (EAT) and lower biventricular function were observed in patients with T2DM than in the controls [69]. In patients with T2DM, EAT volume was correlated with biventricular longitudinal strain and strain rate [70]. This finding indicates that due to the systemically mediated nature of DM, biventricular dysfunction can occur simultaneously or sequentially.

## 5 EVALUATION OF THE SUPERIMPOSED EFFECTS OF T2DM AND COMORBIDITIES ON MYOCARDIUM BY CMR-FT

As mentioned above, CMR-FT is widely used in the evaluation of heart function in diseases, such as DM and hypertension. However, T2DM is often accompanied by other diseases, such as hypertension, coronary heart disease, and dilated cardiomyopathy. A quantitative assessment of the superimposed effect on the myocardium of T2DM and its complications is essential.

### 5.1 Study on Strain in Patients with T2DM and Hypertension

**Fig. (6):**
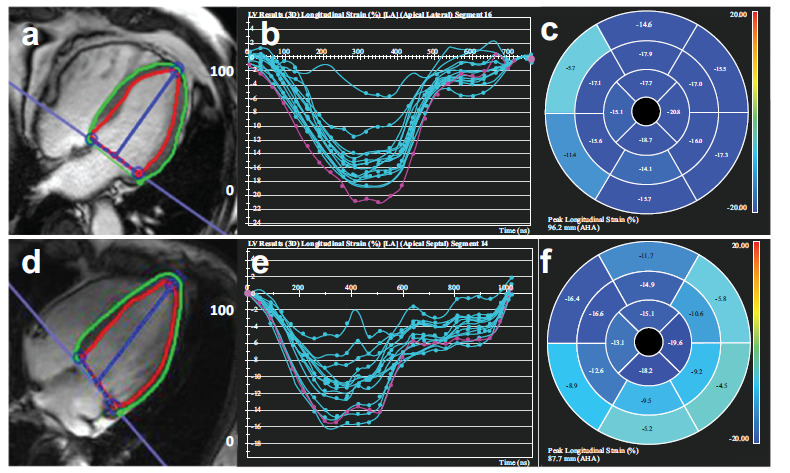
The CMR-derived LV GLS among T2DM patients with and without HT. (**a-c**), T2DM patient, male, 57 years old. (**d-f**), T2DM patient with HT, male, 58 years old. LV four-chamber cine sequence images (**a,d**), LV global longitudinal strain curve (**b,e**), pseudo color maps of LV global longitudinal strain (**c, f**) (**Source:** Figure prepared by the author using the CVI42 post-processing software).

Due to the common risk factors, essential hypertension (HT) is one of the most common complications of T2DM. Approximately 70% of patients with T2DM have HT, and the probability of developing T2DM is approximately 2.5 times greater in patients with hypertension than in a normal group. Compared to normal controls, the coexistence of both resulted in a four-fold increase in the risk of cardiovascular death [71]. The study found that the GLS, GCS, and GRS of LV were lower in patients with T2DM with hypertension than in those without hypertension, and there was no significant difference in LVEF and LV morphology between the two groups (Fig. **6**). Research on biventricular strain found that the GLS of both ventricles and segmental strain of IVS were significantly lower than those in the T2DM group, suggesting that T2DM aggravates ventricular systolic dysfunction in hypertensive patients [71].

Shao *et al*. found that, compared to that in the control group, the LA strain was significantly higher in the T2DM group with hypertension than in the isolated T2DM group [45]. The reason may be that hypertension and DM have superimposed factors leading to cardiac dysfunction, such as excitation–contraction coupling disorder, metabolic disorder, extracellular matrix remodeling, and abnormal microvascular perfusion. In addition, LA compensatory mechanisms and hypertensive drug effects may have an effect on strain [72, 73]. In the case of T2DM with hypertension and HF with preserved EF (HFrEF), DM is an independent factor associated with LV GLS, GCS, and GRS (GRPS *β* = −0.189, *P* = 0.011; GCPS *β* = 0.217, *P* = 0.005; GLPS *β* = 0.237, *P* = 0.002), and aggravated LV longitudinal dysfunction in patients with hypertension and HFrEF, but it has not been reported to cause significant changes in LV morphology [74, 75]. For patients with HFrEF, both hypertension and T2DM can damage LV function because they have a common pathological mechanism leading to heart injury, which also explains that the patients with HFrEF combined with these two diseases have worse outcomes and need timely and individualized treatment [76].

### 5.2 Study on Strain in Patients with T2DM and Myocardial Infarction

Impaired glucose metabolism often accompanies acute coronary syndrome (ACS). Reportedly, one-third of patients with ACS have DM and impaired glucose tolerance, respectively. In addition, patients with ACS and DM are at high risk of recurrent ischemic cardiovascular events. The data indicate the 7-year risk of myocardial infarction (MI) to be 20.2% in patients with DM and 3.5% in patients without DM [77]. CMR imaging enables a comprehensive assessment of the infarcted myocardium, including volume, shape, and strain. For patients after acute myocardial infarction (AMI), myocardial strain has been shown to have a greater prognostic value than LVEF [78]. Therefore, many studies are currently attempting to better understand the interaction of T2DM and AMI using CMR-FT techniques and to determine the optimal risk-stratification strategy in this high-risk patient population.

A large multicenter study, comparing myocardial strain parameters between patients with AMI and with and without DM, LVEF and LA strain independently predicted adverse events in patients without DM. However, in patients with DM, LV GLS emerged as the only independent predictor of poor prognosis. In patients with DM and LVEF ≥35%, LV GLS and LA reservoir strains provided prognostic value for major adverse cardiovascular events [79]. Thus, we can see that assessment of LV and LA longitudinal strain can optimize a risk-stratification strategy in patients with DM after AMI. Although some studies have shown subtle changes in LV GLS and GCS caused by DM, which may be masked by the stronger effect of AMI; however, the added value of strain is still superior to LVEF [80].

### 5.3 Study on Strain in Patients with T2DM and Non-ischemic Cardiomyopathy

DM is one of the main causes of HFrEF in non-ischemic cardiomyopathy. Reportedly, the prognosis of patients with non-ischemic dilated cardiomyopathy (NIDCM) is worse for those complicated with T2DM than for those without T2DM [81]. When patients with NIDCM have abnormal glucose tolerance, the myocardium takes up and biochemically reduces non-esterified fatty acids, and the utilization rate of glucose increases, especially when the heart rate increases. Therefore, some studies have found that the production of myocardial reactive oxygen species is five times higher in patients with DM and NIDCM than in patients with NIDCM alone. In addition, hyperglycemia can lead to blood volume overload, leading to disruption of the natriuretic peptide system activation. Histologically, analysis of endomyocardial specimens from patients with NIDCM and T2DM showed that they had worse myocardial relaxation, increased myocardial fibrosis, and more severe myocardial mitochondrial degeneration [82]. These mechanisms synergistically increase cardiac stress.

A study found LV GLS to be significantly lower in patients with NIDCM and T2DM than in patients with NIDCM but without T2DM. The occurrence of DM was correlated with impairment of LV GLS in patients with T2DM and NIDCM. Another study found DM to be an independent determinant of decreased LV GCS and LV GLS in patients with NIDCM and T2DM, and the LV PS gradually decreased with increasing HbA1c levels [83]. A study on 102 patients with NIDCM by Sakakibara *et al*. showed the prognosis to be worse for the patients with T2DM than for those without T2DM, and multivariate analysis showed T2DM to be significantly associated with an increased incidence of cardiac events. These findings suggest a detrimental effect of T2DM on LV deformation in patients with NIDCM and emphasize the importance of blood glucose control and management in patients with NIDCM and T2DM [43, 84, 85]. Under the guidance of strain, the rational medication with antihyperglycemic and cardioprotective drugs can prevent the progression of HF in patients with NIDCM and T2DM.

### 5.4 Study on Strain in Patients with T2DM and Valvular Disease

Functional mitral regurgitation (FMR) is the most common heart valve disorder in patients with DM and may increase mortality in this population. A DM study showed that about 32% of patients with T2DM had FMR. The all-cause mortality was 3.3 times higher for patients with DM and mild FMR than for patients with T2DM and without FMR, whereas for patients with moderate-to-severe FMR and T2DM, there was a 5.1-fold increase in all-cause mortality [46]. The mechanism of FMR in diabetic cardiomyopathy is as follows: DM can cause LV remodeling and enlargement, and then secondary mitral annulus dilation, papillary muscle displacement, and mitral valve insufficiency. After mitral valve regurgitation occurs in patients with DM, the preload of the LV will further increase, resulting in a decrease in LV systolic function. In addition, T2DM combined with FMR can cause blood to flow back to the LA, resulting in impaired LA compliance, and further impaired LV function [86].

**Fig. (7):**
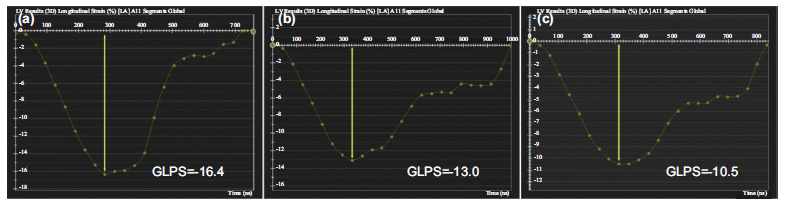
LV global peak strain curve in the longitudinal direction. (**a**), a patient of control group; (**b**), a T2DM patient; (**c**), a T2DM patient with aortic regurgitation. **Abbreviations:** GLPS, global longitudinal peak strain (**Source:** Figure prepared by the author using the CVI42 post-processing software).

A study found that the radial, circumferential, and longitudinal strain of LV in patients with T2DM and FMR decreased more severely. With the aggravation of reflux, the strain of LV also gradually decreased. Diastolic dysfunction was the main symptom in the mild and moderate groups, and the systolic function was further impaired in the severe group. In the correlation analysis, the degree of mitral regurgitation was independently correlated with the radial, circumferential, and longitudinal PS of the LV. In a further study on atrioventricular coupling, mitral regurgitation impaired both LV and LA function in patients with DM [87]. A similar mechanism was found in a study on patients with T2DM and aortic regurgitation (Fig. **7**) [88].

### 5.5 A Study on Strain in Patients with T2DM and Abnormal Metabolism

Chronic kidney disease (CKD), one of the common comorbidities in patients with T2DM, is associated with an increased risk of cardiovascular disease, infection, reduced quality of life, and premature death. Pathology has shown that DM-related metabolic abnormalities, such as hyperglycemia, lead to myocardial extracellular matrix deposition, oxidative stress, and chronic inflammation [89, 90]. Anemia is also a worrying clinical disease when it is combined with T2DM; the prevalence of anemia is as high as 33%, and the cardiovascular system may suffer double damage from T2DM and anemia [91]. In addition, many other risk factors, such as obesity, dyslipidemia, and abnormal thyroid hormones, would synergistically damage the heart when combined with T2DM [92, 93].

The study found that in patients with T2DM and CKD, renal insufficiency aggravated the damage of LV strain, and the estimated glomerular filtration rate (eGFR) and uric acid were independently associated with LV radial, circumferential, and longitudinal PS. This suggests that PS may gradually decrease with worsening eGFR and accumulation of uric acid [94]. Patients with T2DM and anemia had the lowest LV global strain and highest LV mass, whereas the LV concentricity index did not show a significant difference from that of the controls and the isolated patients with T2DM [91, 95]. A recent study reported that total cholesterol (TC) and low-density lipoprotein cholesterol (LDL-C) levels were lower in patients with T2DM hospitalized for a first MI than in patients without (4.4 ± 0.9 *vs*. 5.4 ± 1.2 mmol/l for TC and 2.5 ± 0.8 *vs*. 3.4 ± 1.1 mmol/l for LDL-C, *P* < 0.001 for both comparisons) [96, 97]. Myocardial microvascular function gradually declined with increasing body mass index in both DM and non-DM status [98]. These findings may be explained by the implementation of preventive therapeutic strategies in patients with T2DM, and they also highlight the need for CMR-FT monitoring in these high-risk patients.

In summary, the mechanism and effect of T2DM and its complications on the heart are complex. It is essential to use CMR-FT to comprehensively evaluate the cardiac function of such patients, especially the left atrioventricular and biventricular interaction effects. It can provide patients with accurate information about heart damage and guide clinicians to formulate good treatment strategies [99].

## 6 DISCUSSION

The global longitudinal and circumferential strains of LV have been repeatedly proven to be stable indices to reflect myocardial dysfunction in T2DM patients with or without comorbidities *via* CMR-FT. The global longitudinal strain of LA can serve as an important supplement to LV parameters, with high sensitivity but poor repeatability. The study on atrioventricular and biventricular interaction is new currently. It can compensate for the instability of single chamber indices and also explore the relationship and stability of strain parameters of various chambers. Of course, CMR-FT technology has certain limitations. There are currently various post-processing software for CMR-FT technology, resulting in significant variations in the measured strain values between studies. In addition, it has not yet been widely applied in clinical practice as a routine testing method. Further research and extensive clinical trials are needed to provide a unified reference standard [100, 101].

## CONCLUSION

Myocardial injury associated with T2DM is characterized by diastolic dysfunction with preserved LVEF. CMR-FT can evaluate the subclinical dysfunction of LV, LA, and RV, and allow exploration of the coupling effect between the atrium and ventricle in patients with T2DM. The additive effect of T2DM and its complications on the heart can also be accurately evaluated in the early stage. In addition, with the development and application of radiomics and artificial intelligence, the time resolution and reproducibility of CMR-FT will be continuously improved. Thus, this article highlights that the CMR-FT technique can provide timely and accurate information for the diagnosis of myocardial injury in T2DM patients.

## LIST OF ABBREVIATIONS

T2DM: Type-2 Diabetes Mellitus
HF: Heart Failure
LVEF: Left-Ventricular Ejection Fraction
PS: Peak Strain
